# A Compact Optical MEMS Pressure Sensor Based on Fabry–Pérot Interference

**DOI:** 10.3390/s22051973

**Published:** 2022-03-03

**Authors:** Yonghong Qi, Minghui Zhao, Bo Li, Ziming Ren, Bing Li, Xueyong Wei

**Affiliations:** 1 State Key Laboratory for Manufacturing Systems Engineering, Xi’an Jiaotong University, Xi’an 710049, China; qiyh1314@stu.xjtu.edu.cn (Y.Q.); minghui199402@stu.xjtu.edu.cn (M.Z.); L.B2020@stu.xjtu.edu.cn (B.L.); monol123@stu.xjtu.edu.cn (Z.R.); lb@mail.xjtu.edu.cn (B.L.); 2 State Key Laboratory of Applied Optics, Changchun Institute of Optics, Fine Mechanics and Physics, Chinese Academy of Sciences, Changchun 130033, China

**Keywords:** Fabry–Pérot (FP), pressure sensor, MEMS, diaphragm

## Abstract

Pressure sensors have important prospects in wind pressure monitoring of transmission line towers. Optical pressure sensors are more suitable for transmission line towers due to its anti-electromagnetic interference. However, the fiber pressure sensor is not a suitable choice due to expensive and bulky. In this paper, a compact optical Fabry–Pérot (FP) pressure sensor for wind pressure measurement was developed by MEMS technology. The pressure sensor consists of a MEMS sensing chip, a vertical-cavity surface-emitting laser (Vcsel), and a photodiode (PD). The sensing chip is combined with an FP cavity and a pressure sensing diaphragm which adopts the square film and is fabricated by Silicon on Insulator (SOI) wafer. To calibrate the pressure sensor, the experimental platform which consists of a digital pressure gauge, a pressure loading machine, a digital multimeter, and a laser driver was set up. The experimental results show that the sensitivity of the diaphragm is 117.5 nm/kPa. The measurement range and sensitivity of the pressure sensor are 0–700 Pa and 115 nA/kPa, respectively. The nonlinearity, repeatability, and hysteresis of the pressure sensor are 1.48%FS, 2.23%FS, and 1.59%FS, respectively, which lead to the pressure accuracy of 3.12%FS.

## 1. Introduction

Wind disaster is one of the most terrifying disasters in nature [1]. The survey shows that the losses caused by wind disasters account for more than 50% of all losses caused by natural disasters. When the wind disaster occurred, the damage of the transmission line tower caused a power outage, which greatly affected the reconstruction work [2]. The statistics show that the losses are mainly the damage and collapse of the engineering structure caused by the wind load. When the wind speed reaches 30 m/s, the wind pressure is about 500 Pa. Therefore, the monitor of the wind pressure is necessary to ensure the stable operation of the transmission line tower [3], and the pressure sensor can be selected to monitor the wind pressure. At present, the pressure sensors are divided into the types of piezoresistive [4], piezoelectric [5], capacitive [6], resonant [7], and optical pressure sensors [8] according to the detection method. Among them, piezoresistive, piezoelectric, resonant, and capacitive pressure sensors are popular due to their simple structure and low cost [9,10,11,12]. However, these sensors are easily affected by electromagnetic interference and are not suitable for wind pressure monitoring of transmission line towers [13,14]. In contrast, the optical pressure sensor is immune to electromagnetic interference as the advantage of its optical sensing mechanism [15,16].

In recent years, Fabry–Pérot sensors based on MEMS technology have developed rapidly, and the technology has become mature. It is widely used in the measurement of [1] pressure. However, most of the Fabry–Pérot sensors are optical fiber interference type, and their specific structure is all-fiber (the structure is directly processed in the fiber core) or fiber with MEMS structure. Zhou Ning et al. proposed a fiber optic pressure sensor based on reflective intensity modulation [17]. The sensor consists of two multimode optical fibers with a spherical end, a quartz tube with dual holes, a diaphragm, and a high borosilicate glass substrate (HBGS). The pressure sensitivity of the sensor is about 0.139 mV/kPa. Wang Xue et al. reported a pressure sensor based on fiber Fabry–Pérot (FP), which uses a polarization low-coherence interference demodulator to convert the loading pressure on the silicon diaphragm into cavity length information [18]. It shows a relatively linear response within the pressure range of 3 kPa to 283 kPa, which with a sensitivity of 23.63 nm/kPa. Vengal Rao Pachava et al. designed a metal diaphragm pressure sensor based on wavelength demodulation [19]. The sensor bonds the end of the fiber Bragg grating and the center of the thin-film metal together to eliminate the signal averaging effect. The wavelength shift sensitivity is 32 pm/kPa. Liu Bin et al. researched a Fabry–Pérot based optical fiber acoustic sensor, which uses the phase-generated carrier to demodulate cavity length information. The phase sensitivity is about 10–7 rad/μPa @2 kHz [20]. In application, the optical fiber interferometric sensor needs to build a detection system composed of a benchtop laser, optical fiber coupler, photoelectric detector, etc. The whole detection system is bulky and expensive, with poor practicability, which is not in line with the development direction of miniaturization and integration of sensors [21,22]. In recent years, with the development of integrated micro-optics technology, researchers have begun to explore the development of integrated optical MEMS acceleration sensors. The MEMS chips, laser chips, collimating mirrors, beam splitters, and photodiodes are integrated in a miniature housing [23]. However, there are few reports about integrated optical MEMS pressure sensors.

In view of this, an integrated optical MEMS pressure sensor is proposed in this paper. The sensing chips, laser chips and photodiodes are integrated in a miniature housing. The laser and photodiode form a measurement system to measure the sensitive chip through space, which reduces the coupling loss. The design and mechanism of the optical pressure sensor are first discussed in detail. Then, the fabrication and package of the pressure sensor chip were introduced. Afterward, the performance calibration is performed to characterize the performance of the pressure sensor, and the experimental results show that the sensor has a pressure accuracy of 3.12%FS within a measurement range of 700 Pa. Finally, the paper is summarized with conclusions.

## 2. Design and Mechanism

### 2.1. Working Mechanism

**Figure 1:**
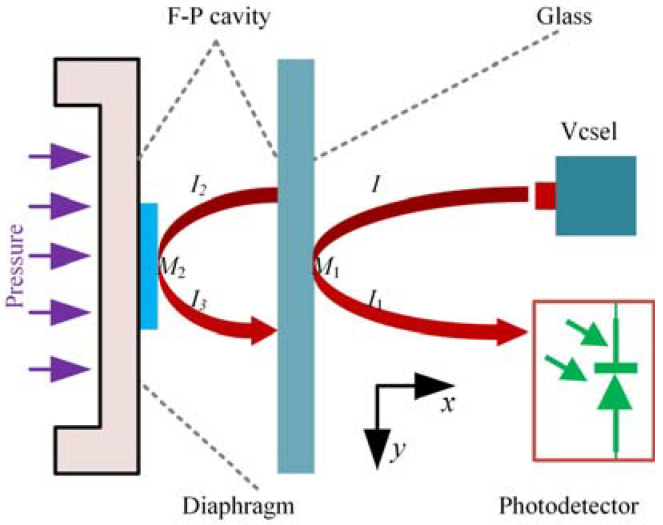
Schematic diagram of the optical cavity of the Fabry–Pérot pressure sensor.

The pressure sensor designed in this paper is mainly composed of the diaphragm, Vcsel, PD, and FP cavity, as shown in Figure 1. The FP cavity is composed of a fixed mirror (glass) and a movable mirror (diaphragm). After the light *I* emitted by Vcsel reaches the surface of the fixed mirror *M*_1_, a part of the light *I*_1_ = I*R is reflected, and the other part of the light *I*_2_ = *I* − *I*_1_ passes through the fixed mirror and reaches the diaphragm (*M*_2_). The light *I*_3_ = *I*_2_*R reflects from *M*_2_ and passes through the fixed mirror *M*_1_, and then interferes with the light wave *I*_1_. The intensity of the interference is related to the cavity length of the FP cavity. When the diaphragm is subjected to the loaded pressure, as shown in Figure 1, the diaphragm will be deformed across the *x*-direction. The deflection changes the cavity length of the FP cavity and then changes the intensity of the interference. The PD is used to monitor the light intensity, and the loaded pressure can be calculated according to the light intensity.

### 2.2. Pressure Sensing Diaphragm

The diaphragm structure can be modeled as a thin membrane with four clamping edges. When the membrane is loaded with uniform pressure, the maximum deflection of the membrane is less than one-fifth of the membrane thickness [24], and the deflection of the membrane can be expressed as [25]:

(1) $$\omega_{x,y}=\frac{49Pa^{4}}{2304D}{\left(1-{\left(\frac{x}{a}\right)}^{2}\right)}^{2}{\left(1-{\left(\frac{y}{a}\right)}^{2}\right)}^{2}$$

where 2*a* is the side length of the membrane, *P* is the loaded pressure, and *D* is the flexural rigidity, which can be expressed as:

(2) $$D=\frac{Eh^{3}}{12\left(1-v^{2}\right)}$$

where *h* is the thickness of the membrane, *E* is Young’s modulus, and *v* is the Poisson’s ratio.

**Figure 2:**
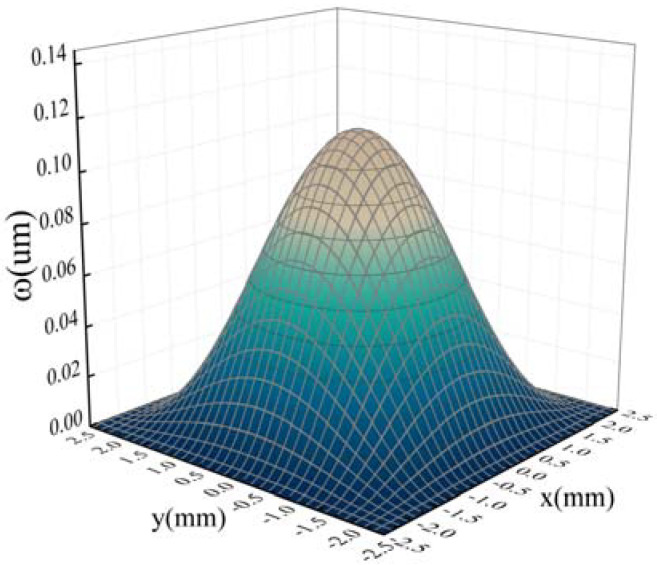
The deflection of the thin membrane.

The deflection of each point on the membrane can be calculated by Equation (1) and the results are shown in Figure 2 (*a* = 2.5 mm, *h* = 100 μm, *P* = 2 KPa, *Ε* = l60 GPa, *v* = 0.22).

**Figure 3:**
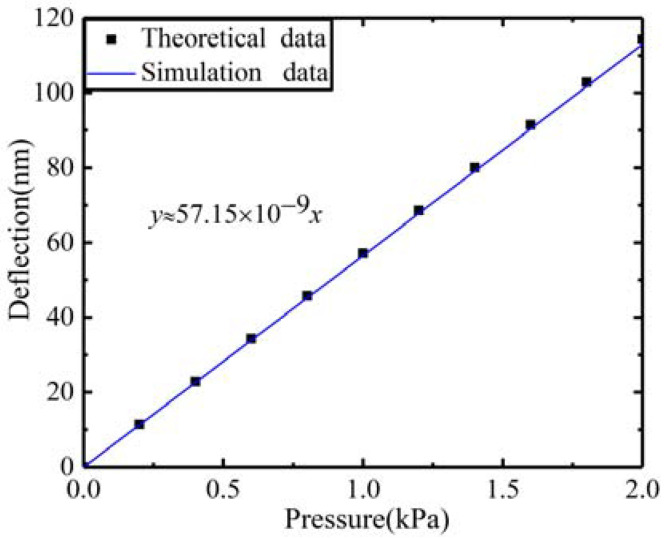
The maximum deflection when loaded pressure.

To verify the feasibility of the theoretical analysis, a finite element model was developed in COMSOL software. The diaphragm is modelled as a square thin membrane with four clamped edges for achieving high quality mesh and convergence of the solution. The model is meshed using triangular elements, and the minimum size of triangular elements is 1.57 × 10^−2^ mm. The relationship between the maximum deflection and the loaded pressure is shown in Figure 3. It can be seen that the theoretical data and the simulation data are in good agreement, and the sensitivity of the membrane is *S*_m_ = 57.15 nm/kPa.

Therefore, the maximum deflection of the thin membrane can be expressed as:

(3) $$\omega_{\max}=\frac{49a^{4}}{2304D}P=S_{m}\cdot P=57\times 15\cdot 10^{-12}\cdot P$$

### 2.3. FP Cavity

**Figure 4:**
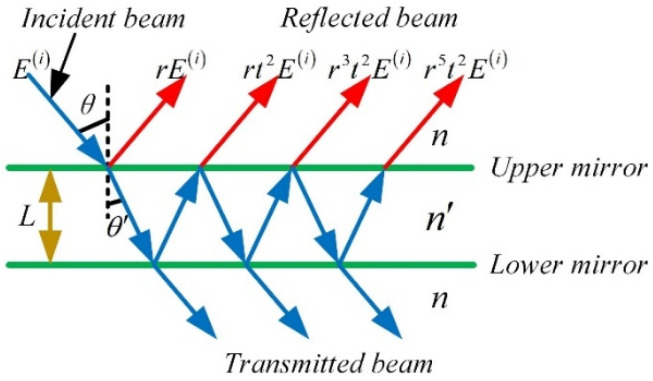
Schematic diagram of multi-beam interference in plate.

The FP interference cavity can be equivalent to plate interference, as shown in Figure 4. Consider the refractive index of the parallel plate and the surrounding medium is *n*′ and *n*, respectively. Suppose a beam of the monochromatic plane light wave was inputted on the plate with an angle *θ*. $E^{\left(i\right)}$ is the electric vector amplitude of the incident wave, which is a complex number. *t* and *r* are the transmission coefficient and reflection coefficient of the plate, respectively.

Superimposing the reflected waves from the upper mirror, the intensity of the reflected wave can be expressed as:

(4) $$I\left(r\right)=\frac{2\left(1-\cos\left(\delta \right)\right)R}{1+R^{2}-2R\cos\delta }I^{\left(i\right)}$$

where, $I^{\left(i\right)}$ is the intensity of incident light. δ is the phase difference between two adjacent reflected beams, and its expression is:

(5) $$\delta =\left(\frac{2\pi }{\lambda }\right)2nL\cos\theta$$

when the reflectivity *R* is very small, substituting the Equation (5) into the Equation (4), the relationship between the interference intensity and the cavity length can be described as:

(6) $$I\left(r\right)=2R\left[1-\cos\left(\frac{4\pi nL\cos\theta }{\lambda_{0}}\right)\right]I^{\left(i\right)}$$

When the pressure load on the diaphragm, the displacement of the diaphragm will change cavity length. The expression of cavity length after pressure loaded is:

(7) $$L=L_{0}-\omega_{\max}=L_{0}-S_{m}\cdot P$$

where *L*_0_ is the initial cavity length of the FP cavity.

**Figure 5:**
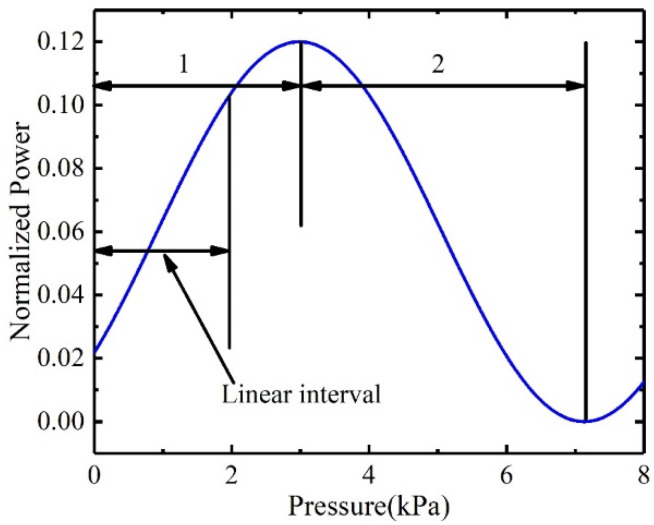
The output power of the pressure sensor under the loaded pressure.

Substituting Equation (7) into Equation (6), the relationship between the light intensity and the external loading pressure can be obtained. The results are shown in Figure 5. (i.e., $\lambda_{0}=940\;\mathrm{nm},\;\theta =7.12^{\circ },\;R=0.03,\;L_{0}=25\;\mu m,\;n=1$).

(8) $$I=\frac{I\left(r\right)}{I^{\left(i\right)}}=2R\left[1-\cos\left(\frac{4\pi n\cos\theta }{\lambda_{0}}\left(L_{0}-S_{m}\cdot P\right)\right)\right]$$

Figure 5 shows that the interference intensity from the FP cavity changes sinusoidally with the increase of external loading pressure. The effective working range of the pressure sensor is half the period of the sine curve, otherwise which the same output value will correspond to different loaded pressures. The working range of the pressure sensor can be interval 1, interval 2, or some other sub-sequent interval (e.g., when the initial loading pressure is 0 kPa, the working interval of the pressure sensor is interval 1; when the initial loading pressure is 6 kPa, and only the relative pressure value needs to be measured, the working range of the pressure sensor is interval 3). The maximum effective working range of the pressure sensor is 4.11 kPa. The pressure sensor works in the linear interval of the working range. The chopped region of the interval 1 is not in the linear interval and therefore has no effect on the actual measurement range. The linear working range of this sensor in the application is from 0 to 2 kPa, which meets the wind pressure monitoring requirements of the transmission line tower.

## 3. Fabrication and Packaging

### 3.1. Fabrication of the Diaphragm

**Figure 6:**
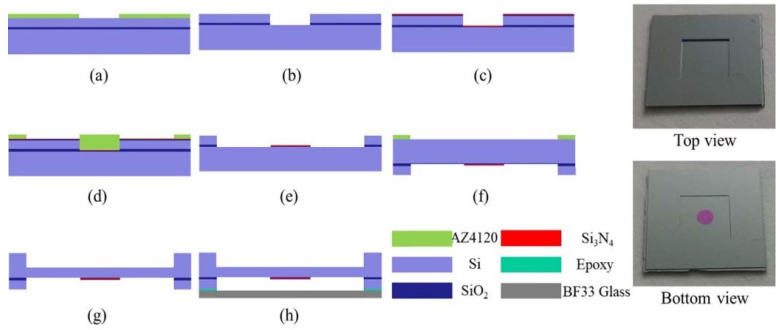
The fabrication process and photos of the fabricated pressure sensor: (**a**) Lithography; (**b**) DRIE (Deep Reactive Ion Etching); (**c**) Deposits SiO_2_ and Si_3_N_4_; (**d**) Lithography; (**e**) Wet etching; (**f**) Lithography; (**g**) DRIE (Deep Reactive Ion Etching); (**h**) Anodic bonding.

The diaphragm chip designed in this paper was fabricated by SOI (device: 25 μm, oxide: 1 μm, handle: 300 μm) and glass wafer, as shown in Figure 6. Firstly, the device layer was etched to the oxide layer by DRIE to form an FP interference cavity. The BOE solution was used to etch silicon oxide, as shown in Figure 6a,b, followed by the 320 of SiO_2_ and 147 nm of Si_3_N_4_ being deposited on the bottom of the diaphragm. (By depositing SiO_2_ and Si_3_N_4_, the reflectivity at the bottom of the diaphragm was about 0.03, as shown in Figure 6c.) Then the device layer was etched to the oxide layer by DRIE to form the bottom of the diaphragm, and the BOE solution was used to etch silicon oxide, as shown in Figure 6d,e. Afterward, the handle layer was etched 200 μm by DRIE to form a diaphragm with a thickness of 100 μm and a side length of 5 mm. Finally, the diaphragm and the BF33 glass wafer (the reflectivity is about 0.03) were glued together with epoxy to form an FP-sensing chip with a cavity length of 25 μm, as shown in Figure 6f–h.

### 3.2. Packaging of the Sensor Chip

**Figure 7:**
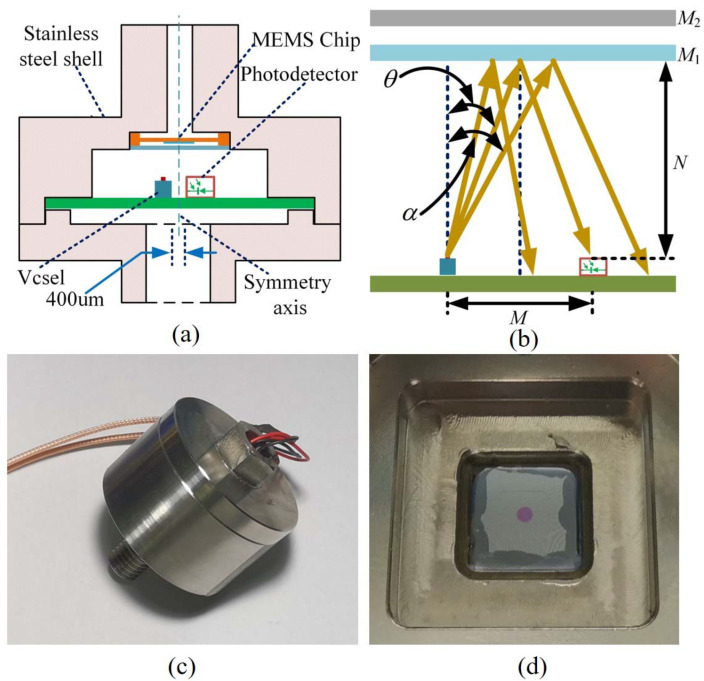
(**a**) The schematic diagram of the stainless-steel package, (**b**) the schematic diagram of optical path, (**c**) the stainless-steel package, (**d**) the pressure sensor installation.

A stainless-steel package was designed and fabricated to be convenient for the performance test of the pressure sensor, which is shown in Figure 7c. The MEMS sensor chip was first positioned on the package by a four-point fixation method under the microscope and then sealed with epoxy as shown in Figure 7d. Then the Vcsel and photodiode (LP000E) horizontally were fixed under the chip. The horizontal distance between the Vcsel and the photodiode was 600 μm, and the distance between the Vcsel and fixed mirror *M*_1_ was 2.4 mm. The Vcsel and photodiode were symmetric about the symmetry axis of the diaphragm, as shown in Figure 7a,b. By Equation (9), the value of *θ* was 7.13 deg.

(9) $$\theta =\arctan\left(\frac{M}{2N}\right)$$

## 4. Performance Calibration

**Figure 8:**
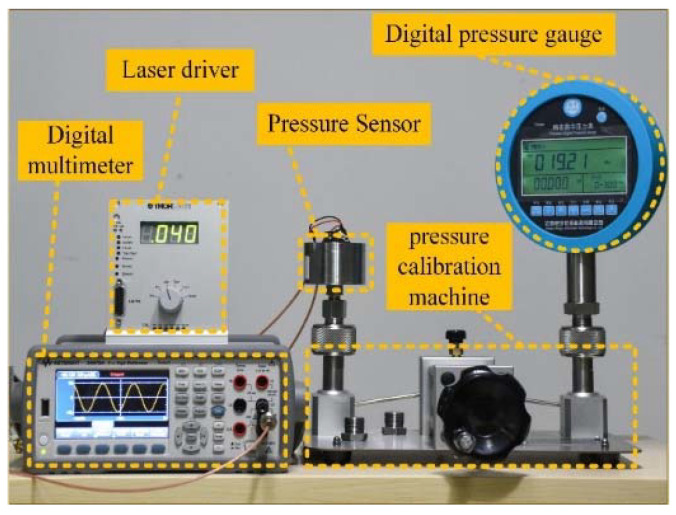
The pressure sensor performance test platform.

The experimental platform is shown in Figure 8, which includes the pressure sensor, a digital pressure gauge, a pressure calibration machine, a digital multimeter, and a laser driver. The pressure sensor and digital pressure gauge were installed on the manually actuated air pressure calibration machine. The accuracy and measurement range of the digital pressure gauge was ±0.02%FS (full-scale) and 300 kPa, respectively. The internal pressure of the manually actuated air pressure calibration machine was controlled by a hand-turned piston and displayed on the digital pressure gauge. The THORLABS-ITC102 laser driver was used to drive Vcsel with a constant current of 8 mA, and the output of the sensor was acquired by digital multimeter 34470A (Keysight, Santa Rosa, California, United States). In the test, the pressure applied to the diaphragm was gradually increased from 0 to 5 kPa with a step of 200 Pa. The pressure was related to the compressed volume and temperature of the air, so the adjusted pressure would gradually decrease and then stabilize. Before collecting data, we waited for a long time until the pressure was stable. The output of the sensor in each pressure setpoint was collected 10s’ data with a sampling frequency of 2.5 Hz.

**Figure 9:**
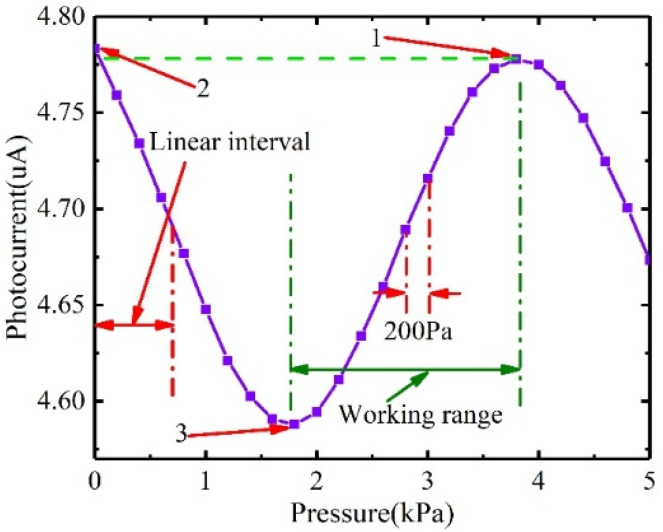
The output photocurrent value of the pressure sensor vs. the loading pressure.

**Figure 10:**
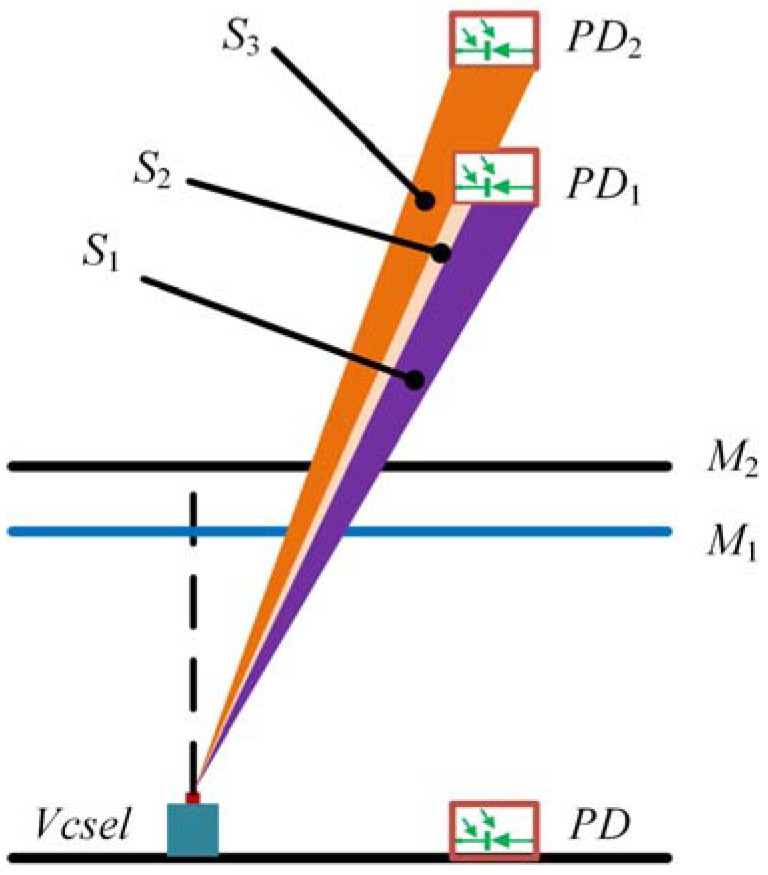
Geometry of the light intensity modulation mechanism.

The output photocurrent of the sensor with the increase of applied loaded pressure is shown in Figure 9. As the increases of loaded pressure, the photocurrent changes sinusoidally, which is consistent with the changing trend described in Figure 5. However, the minimum value of the Equation (8) was zero, but the minimum value of the experimental data was not zero. This suggests that only part of the light emitted from the Vcsel was interfered, and most of the light was directly reflected on the PD. The reason for the significant difference between Figure 5 and Figure 9 is that Figure 9 had a DC bias. The intensity received by the PD was mainly from the reflection of the fixed mirror *M*_1_ and the movable mirror *M*_2_ [26], as shown in the Figure 10. The PD1 and PD2 were images of PD with respect to fixed mirror *M*_1_ and movable mirror *M*_2_, respectively. The light intensity reflected by the fixed mirror *M*_1_ and the movable mirror *M*_2_ was *S*_2_ + *S*_1_ and *S*_2_ + *S*_3_, respectively. Interference can occur due to the correlation of *S*_2_. The intensity received by the PD was 2 × *S*_2_ + *S*_1_ + *S*_3_. Since *S*_1_ and *S*_3_ had no correlation, *S*_1_ + *S*_3_ was the received intensity bias. In the optical system, the *S*_1_ + *S*_3_ was much larger than *S*_2_; therefore, the amplitudes in Figure 5 and Figure 9 differed significantly.

Figure 5 shows that the maximum effective working range of the sensor is 4.11 kPa, while Figure 9 shows that the maximum effective working range of the pressure sensor was 2 kPa. This is mainly caused by the processing error of the sensing chip. Equation (8) shows that when the sensor output from the minimum point 3 to the maximum point 1, the cavity length should satisfy the relationship follow:

(10) 4πnΔLλ0=π⇒ΔL=λ04

The wavelength of the Vcsel used in this paper is 940 nm. When the output of the sensor changes from point 3 to 1, the cavity length changes 235 nm. Figure 9 shows that the pressure difference between point 3 and 1 is 2 kPa. When the maximum deflection was less than one-fifth of the diaphragm thickness, the deformation of the diaphragm had a linear relationship with the loaded pressure. Therefore, the sensitivity of the diaphragm designed and processed in this paper was 117.5 nm/kPa. This was bigger than the designed sensitivity value of 57.15 nm/kPa. It is because of the existing of processing error, which makes the diaphragm thinner than the design. Substituting Equation (2) into Equation (3), Equation (11) can be obtained. Equation (11) shows that the sensitivity of the diaphragm is inversely proportional to the thickness, the thinner the film, the greater the sensitivity. The thickness of the sensitive film has no effect on mechanical thermal noise. However, the thickness of the sensitive film needs to ensure that the maximum deflection is within the working range. Equation (8) shows that the output curve of the pressure sensor is a sine curve, and the effective working range of the sensor is half of the period. The effective working range of the sensor is shown in Equation (12). Equation (12) shows that the effective working range of the pressure sensor is inversely proportional to the sensitivity of the diaphragm: The greater the sensitivity, the smaller the working range. Therefore, the processing error can increase the sensitivity of the diaphragm and reduce the effective working range of the sensor.

(11) $$S_{m}=\frac{49a^{4}12\left(1-v^{2}\right)}{2304Eh^{3}}$$

(12) $$T_{range}=\frac{T}{2}=\frac{2\pi }{2\omega }=\frac{\lambda_{0}}{4S_{m}}$$

Figure 5 also shows that the output value of the sensor at 0 kPa was approximately the average value of the peak-to-peak value of the output curve. However, Figure 9 shows that the output value of the sensor at 0 kPa was approximately equal to the maximum value. The actual output curve of the sensor had a phase shift compared to the ideal output. This was mainly caused by the bonding error of the Fabry–Pérot cavity. In this paper, epoxy was used to connect the sensor chip and the glass to form an FP cavity. The length of the FP cavity after bonding would be larger than the ideal design, as the thickness of epoxy cannot be zero. Equation (5) shows that the phase difference between the two light beams increases linearly with the increase of the cavity length. Therefore, there was a fixed phase difference between the actual output curve and the ideal curve. Figure 9 shows that the extreme value at point 1 was lower than the value at point 2. This shows that the output of the sensor has a downward trend as time increases. This deviation was caused by temperature changes of Vcsel. The output power of Vcsel decreased with increasing temperature.

**Figure 11:**
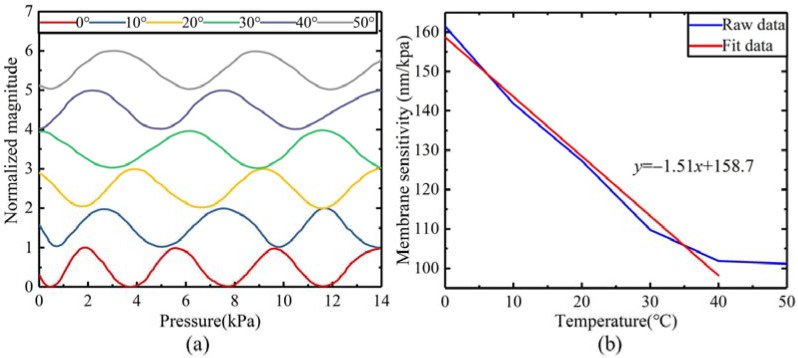
(**a**) Pressure responses of the sensor at different temperatures; (**b**) pressure sensitivity curve at different temperatures.

Figure 9 also shows that the effective working range of the pressure sensor can be the range between point 2 and 3 or point 3 and 1 (the range between adjacent extreme points). The pressure difference inputted by the pressure sensor in practical applications started from 0 kPa (the pressure difference between the environment and the FP cavity). Therefore, the effective working range of the sensor was 0~1.75 kPa. However, this range was not a linear interval, and the output of the sensor had a non-linear relationship with loaded pressure. To better calibrate the output characteristics of the sensor, a range between 0 and 0.7 kPa was selected as the actual working interval to recalibrate. The effective range of the cavity length measurement of the pressure sensor was 1/8 of the wavelength of the Vcsel. The tunable laser [27] and white-light interferometric demodulation algorithm [28] could be used to enhance the range. We applied incremental pressure to the pressure sensor at different temperatures, normalized the output data, and added a bias value, as shown in Figure 11a. As the thermal expansion coefficient, Young’s modulus and Poisson’s ratio and mechanical parameters of Si material were affected by temperature, and the pressure sensitivity increased with the temperature with a coefficient of 1.51 nm/(KPa°C), as shown in Figure 11b.

**Figure 12:**
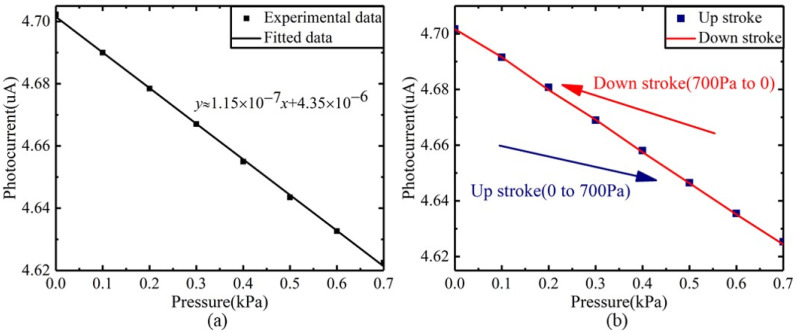
(**a**) The nonlinear of the sensor, (**b**) the hysteresis and repeatability of the sensor.

In the experiment, the pressure applied to the diaphragm was gradually increased from 0 to 0.7 kPa with a step of 100 Pa. The output of the sensor in each pressure setpoint was collected 20s’ data with a sampling frequency of 2.5 Hz. To eliminate the noise of the loaded pressure, the data under each loaded pressure is averaged, and the experimental data is shown in Figure 10. A linear fitting was performed on the data between 0 and 0.7 kPa, and the fitting result is shown in Figure 12a. The results show that the raw data fluctuates from the fitted data. Besides the influence of the output characteristics of the FP interferometric cavity, two other factors may contribute to this fluctuation. The first factor is the temperature, which has been explained above, and the second is the influence of environmental pressure. The fluctuation of environmental pressure causes the fluctuation of loaded pressure, which makes the output fluctuate. Figure 12a indicates that the output sensitivity of the pressure sensor is 115 nA/kPa. The experimental results of repeatability and hysteresis are shown in Figure 12b. After calculation, the nonlinearity, repeatability, and hysteresis of the sensor are 1.48%FS, 2.23%FS, and 1.59%FS, respectively. The pressure accuracy *A* can be calculated by the Equation (13):

(13) $$A=\sqrt{\xi_{H}^{2}+\xi_{L}^{2}+\xi_{R}^{2}}$$

where $\xi_{H},\xi_{L},\xi_{R}$ are the nonlinearity, repeatability, and hysteresis, respectively. After calculation, the pressure accuracy was 3.12%FS, which was lower than the sensors in the market. Although the accuracy of the pressure sensor was lower than the sensors on the market, the pressure sensor designed in this paper is a new concept. At present, most of the products on the market were piezoresistive, resonant, and optical fiber types, but this paper innovatively proposed a micro-integrated optical sensor. Compared with fiber optic pressure sensors, this structure reduces the system volume, and was more conducive to miniaturization and integration. In addition, in theory, optical detection had ultra-high displacement resolution compared with other existing types, which can improve pressure measurement resolution, etc.

The compact Fabry–Pérot pressure sensor designed in this paper had a working range of 0–700 Pa and an accuracy of 3.12%FS, which met the measurement requirements of wind speed (0–30 m/s) [29]. Equation (13) shows that the nonlinearity, repeatability, and hysteresis jointly determined the pressure accuracy. Equation (8) showed that the output of the FP cavity has inherent nonlinear characteristics. The working range of the FP cavity was designed in a region with good linearity, but it cannot eliminate the nonlinearity. There are two approaches to reduce nonlinearity. One approach is to further narrow the working area, so that the working area is located in the region of high linearity. Another approach is to use algorithmic compensation. Algorithm compensation can establish the correspondence between the output and input of the FP cavity, and then compensate for the output of the sensor. This can reduce the sensor’s nonlinear error.

The environment temperature has a great influence on the repeatability and hysteresis of the sensor. Temperature fluctuations affect the output of the Vcsel, which in turn affects the curve between input and output, and then make the deviation of repeatability and hysteresis. The reasons for temperature fluctuations have been discussed above. Two solutions can reduce the temperature drift of the sensor. The first solution is to add a photodiode in the internal optical path to monitor the output power of Vcsel and then use the algorithm to compensate the temperature drift of the sensor. The second solution is to use thermoelectric cooler (TEC) to actively control the temperature and make Vcsel work in a stable environment. The pressure accuracy can be further improved after optimizing.

The comparison between proposed pressure sensor and other reported optical pressure sensors is shown in Table 1. At present, most of the optical pressure sensors use fiber as the transmission medium. In application, the detection system consisting of broadband light source, spectrometer and other instruments needs to be built. The whole system is bulky and expensive. Compared with the pressure sensor that uses optical fiber as the transmission medium, the pressure sensor designed in this paper integrates all the elements in a small chamber and uses the space as the transmission medium.

This paper designed an integrated pressure sensor which is not chip level integrated. The design of integrated pressure sensor was to verify the feasibility of chip level integration. Experimental results showed that chip level integration was feasible. Three steps were required to achieve chip level integration of pressure sensors. First, the Vcsel and PD wafers were bonded to the bottom silicon. Then, the sensing chip was processed on the top silicon. Finally, the bottom silicon and the top silicon were bonded to form a pressure sensor. Chip level integration of pressure sensors has significant advantages in the following areas. On the one hand, control the parallelism of Vcsel and PD through micro manipulation to reduce the loss of light intensity. On the other hand, the cavity length can be accurately controlled by bonding to avoid the drift of the initial operating point.

## 5. Conclusions

In this paper, an optical pressure sensor based on FP was reported. The pressure sensor was integrated by a MEMS sensing chip which consisted of an FP cavity and a pressure sensing diaphragm, Vcsel, and PD. It had the advantages of high accuracy, small size, and ease of integration. First of all, the sensor’s working mechanism and structural design were studied. Then the sensor chip was processed and packaged. Finally, the test platform was built to calibrate the sensor performance. The results show that the sensitivity of the sensor was 115 nA/kPa. The nonlinearity, repeatability, and hysteresis of the sensor were 1.48%FS, 2.23%FS, and 1.59%FS, respectively. The pressure accuracy was 3.12%FS. The measurement range of the pressure sensor was 700 Pa, which met the wind pressure monitoring requirements of the transmission line tower.

## Figures and Tables

**Table 1:** Comparison with other optical pressure sensors.

	Working Range	Transmission Medium	Sensitivity	Demodulation Device
our work	0–700 Pa	Space	117.5 nm/kPa	Vcsel and PD
[30]	0–50 kPa	Fiber	70.5 nm/kPa	Broadband light source and Spectrometer
[21]	2 kPa–7 kPa	Fiber	32.4 µm/kPa	Broadband light source and Spectrometer
[31]	0–10 kPa	Fiber	242 nm/KPa	Broadband light source and Spectrometer

## Data Availability

Data in this paper is available from the corresponding authors upon request.
